# Malignant hyperthermia on ICU – sudden attack of the “snake”

**DOI:** 10.1186/1471-2253-14-S1-A11

**Published:** 2014-08-18

**Authors:** Stephan Johannsen, Susanne Mögele, Norbert Roewer, Frank Schuster

**Affiliations:** 1 Department of Anesthesia and Critical Care, University of Wuerzburg, Wuerzburg, 97080, Germany

## Background

Fulminant malignant hyperthermia (MH) is a rare emergency that should be known to every medical professional although practical exposure is a rarity even for experienced anesthesiologists. The vast majority of documented MH cases occurred during general anesthesia in the operating room following application of volatile anesthetics and/or depolarizing muscle relaxants. With increasing utilization of the anesthetic conserving device AnaConDa® for sedation of intensive care patients, who could benefit from reduced duration of mechanical ventilation and earlier hospital discharge, volatile anesthetics made their way into the intensive care unit (ICU).

## Case report

With oral and written consent of the patient, we report the case of a 59-years old male, who was hospitalized due to persistent lumbalgia. On the second day of his hospital stay, increasing dyspnoe led to ICU admission and required tracheal intubation and mechanical ventilation. Sedation was maintained by propofol and sufentanil. A chest radiograph revealed bilateral pulmonary infiltration, suggestive of influenza pneumonia. Moxifloxacin, piperacillin/tazobactam and oseltamivir were started immediately. When after four days of mechanical ventilation an additional sedative became necessary, the anesthetic conserving device for sevoflurane sedation was installed. Suddenly, following five hours of sevoflurane administration, hemodynamic instability characterized by rapidly dropping arterial blood pressure occurred. Arterial blood gas analysis revealed severe acidosis (pH 7,17; pCO_2_ 70,4 mmHG; pO_2_ 104 mmHG (fiO_2_ 50%), base excess 9,8; lactate 0,6 mmol/l). A rapid increase of body temperature from 39.6 °C to 40.7 °C within 30 min was noticed. Creatine kinase and myoglobin levels were significantly elevated to maximum levels of 3455 U/L and 4197 µg/L due to acute rhabdomyolysis. As soon as MH was suspected, sevoflurane application was discontinued and Dantrolene was administered intravenously. After treatment the hemodynamic and metabolic situation gradually improved. The subsequent ICU stay remained uneventful and the patient was extubated on day 17 without neurological deficits. Nine month after the suspected MH event, the patient underwent muscle biopsy and in-vitro-contracture-testing at the Wuerzburg MH unit. His MH susceptibility was confirmed by significant contractures at the defined thresholds after halothane and caffeine exposure.

## Conclusions

The presented case underlines the significance of MH as differential diagnosis in intensive care patients with hemodynamic and metabolic breakdown, if volatile anesthetics are used for sedation. Hence, awareness of the symptoms of MH and immediate initiation of adequate treatment can be crucial not only in the operating room.

## Note

This abstract was awarded by the EMHG as best clinical presentation.

